# The Levonorgestrel Intrauterine System: A Pragmatic View of an Excellent Contraceptive

**DOI:** 10.9745/GHSP-D-15-00330

**Published:** 2015-12-15

**Authors:** Roy Jacobstein, James D Shelton

**Affiliations:** ^a^​ IntraHealth International, Chapel Hill, NC, USA; ^b^​ Global Health: Science and Practice, Washington, DC, USA

## Abstract

The levonorgestrel intrauterine system (LNG IUS) has major advantages and could be a “game-changer” in improving contraceptive choice and use. It faces important challenges, however, including: (1) high commodity cost; (2) often-strong provider resistance to IUDs and difficult programmatic requirements; (3) need for demand creation, including assessing if markedly reduced menstrual bleeding is attractive to clients; and (4) the many requirements for introducing any new contraceptive. A good next step would be a well-focused and multifaceted “learning introduction” to assess the LNG IUS’s potential in several low-income countries, with rapid scale-up if results are promising.

See related article by Hubacher.

In this issue of *Global Health: Science and Practice*, Hubacher makes a good case for the levonorgestrel intrauterine system (LNG IUS) and why donors should purchase it for provision in African family planning programs.^1^ The LNG IUS is indeed an excellent contraceptive. It is highly effective, with only 2 pregnancies per 1,000 women in 1 year of typical use,^2^ a level of effectiveness 4 times that of the copper-containing IUD, and 35 and 70 times that of the injectable and pill, respectively. The LNG IUS also has very high satisfaction and continuation rates, and it confers important non-contraceptive—even therapeutic—benefits. And, like other long-acting reversible contraceptives (LARCs), it is suitable for all reproductive intentions (delaying, spacing, or limiting births). Both the American College of Obstetricians and Gynecologists and the American Academy of Pediatrics have endorsed LARCs as “first-line” method choices for adolescents and young women.^3^^,^^4^ No wonder the LNG IUS is fueling a rise in IUD use in Europe and the United States. (Prevalence of IUD use is now over 6% in the United States, representing 9% of all modern method use among women aged 15–44.^5^)

The LNG IUS is fueling a rise in IUD use in Europe and the United States.

Hubacher cites 3 obstacles to greater donor interest in the LNG IUS: high commodity cost; the belief that currently available method options are sufficient; and doubt about overcoming the barriers that the copper T 380A IUD (Cu T) often faces. He then advances 6 reasons why international donors should buy the LNG IUS: (1) it is on the World Health Organization’s (WHO’s) Essential Medicines List; (2) it is well accepted worldwide; (3) it has a “unique/advantageous delivery system” and important non-contraceptive benefits; (4) it will “activate” some countries; (5) many women in sub-Saharan Africa want to use intrauterine contraception; and (6) more highly effective options are needed.

To more fully consider Hubacher’s arguments, we address 5 somewhat overlapping aspects:

## 1. Cost Considerations

Costs—absolute commodity cost, commodity cost relative to similar methods (or methods occupying a similar niche), service delivery costs, and opportunity costs—are all, appropriately and necessarily, key considerations for donors, policy makers, and program leaders. Regarding commodity cost, it is relevant to recall that in the early days of implant availability, the public-sector commodity cost (for Norplant) was US$23.80 per set (in 1990 dollars). Today in 2015, thanks to donor volume guarantees to manufacturers, the unit cost of implants has been reduced by two-thirds, to $8.50 per set.^6^ It seems unlikely that the LNG IUS would be available at a unit cost to donors below the 2015 unit cost of implants. By contrast, the cost of the Cu T IUD to donors is as low as $0.35 per device.^6^ The funding implications of these large differentials in respective price points are seemingly also large. On the other hand, it could be argued that the lower commodity cost of the Cu T IUD is somewhat beside the point, because use of the Cu T (or any) IUD is below 1% in the majority of sub-Saharan African countries.^7^ Thus, in most of these countries, the LNG IUS would essentially be a new method for programs, and could be considered as such by policy makers, program leaders, and donors.

It seems unlikely that the LNG IUS would be available to donors below the 2015 unit cost of implants of $8.50 per set.

## 2. Categorization of the LNG IUS

Whether or not—and if so, in what respects—the LNG IUS is an IUD has a number of important dimensions and implications. Hubacher presents a good summary of how the LNG IUS and Cu T IUD differ. But there are also a number of important, programmatically relevant parameters in which the methods are similar. First, both devices require a skilled, unbiased, and motivated service provider in order for clients to receive the method. Second, both require pelvic examination for placement, often a limitation in traditional societies and low-resource settings. Third, both require accurate knowledge by clients—and the Cu T (the IUD generally made available by donors) has long faced a plethora of misunderstandings and myths. Fourth, despite the LNG IUS’s known benefit in reducing abnormally heavy menstrual bleeding and presumed positive effect on anemia, the reduced bleeding or frank amenorrhea it causes could be more problematic in traditional settings where sociocultural beliefs, proscriptions, and/or fears related to menstruation and amenorrhea are more common. None of this means the LNG IUS would not be a very good addition to a country’s method mix; only, rather, that these IUD-related barriers would need to be addressed.

## 3. Entrenched Service Provider Perspectives and Behaviors

Fostering “unlearning” among medical professionals, even when it is “evidence-based,” is often difficult and time-consuming.^8^^,^^9^ Service providers are not “empty vessels” waiting to have their minds filled with new knowledge, which, in turn, will prompt new behavior.^10^ They have their own “truths” and operate on the quite-reasonable general principle that, “What has worked for me has worked, so why change?”^11^ This is a major reason that updated service policies fail to diffuse into everyday service provision and that “research-to-practice” or adoption of “best practices” is generally slow going. Also, what may seem like “lack of provider motivation” is often a response to already-heavy workloads with no compensatory payment or relief from other existing duties in exchange for assuming additional ones. (Inserting IUDs is more time-consuming than provision of implants or short-acting resupply methods like injectables or pills-although removal of implants is more difficult than with IUDs.) Furthermore, in situations of low client demand, it can be difficult for trained providers to maintain their skills.

Changing providers’ specific understandings and behaviors regarding IUD provision can be particularly difficult. In our experience, providers often have exaggerated but deep-seated concerns related to the triad of sexually transmitted infections (STIs), pelvic inflammatory disease (PID), and infertility, as well as misunderstandings regarding eligibility, believing wrongly that a woman needs to be married or to have had a child to be eligible to receive an IUD.^12^ These impediments contribute to the low IUD use seen in a number of sub-Saharan African countries. So even as the LNG IUS is framed programmatically—as it should be—as a new and different method (albeit with the best features of the IUD and the pill), considerable program effort would be needed to ensure that provider biases and client perceptions do not generalize from the Cu T experience.

Considerable program effort would be needed to ensure provider biases and client misperceptions about the Cu T IUD do not spill over into LNG IUS introduction efforts.

A possible programmatic remedy is suggested in Hubacher et al.’s recent study of the perspectives of 27 IUD service providers from Marie Stopes Kenya.^13^ These providers had positive attitudes toward both the Cu T IUD and the LNG IUS, and they behaved accordingly, providing 30,000 IUDs to Kenyan women in 1 year (mainly the Cu T, because of limited LNG IUS commodities, which were provided free by the International Contraceptive Access [ICA] Foundation, described below). Marie Stopes Kenya is a nongovernmental organization, however, and a “closed system” delivering family planning via fixed sites and mobile services (in private-public partnership with the Ministry of Health). It is able to employ family planning-dedicated providers selected not only for their service delivery skills but also for their positive attitudes and commitment to family planning, and then to supervise and reward them accordingly. Generalizability of this approach to the larger and more diffuse and resource-strapped African public sector itself might be limited.

## 4. Uncertain Extrapolation From Recent Experience in Europe and the United States

The recent rapid uptake of the LNG IUS in the United States and Europe may not necessarily be predictive of an LNG IUS trajectory in Africa. High-income countries have a more ample supply of service providers, contraceptive methods and services are much more widely and easily accessible to clients, women are more highly empowered and knowledgeable about their contraceptive options, and bias against the IUD is less (now, as opposed to the situation during the post-Dalkon Shield era of the 1980s and 1990s). Conversely, health workforce and health system constraints in sub-Saharan Africa, particularly for clinical method provision, are unfortunately widespread and problematic, as the recent Ebola outbreak has underscored. This reality is a chief reason that short-acting resupply methods of family planning predominate in the method mix of sub-Saharan African countries.

The recent marked increases in implant use in sub-Saharan Africa may also not be predictive of how the LNG IUS might fare, given the ways that provision of these 2 hormonally based methods differs. The implant needs no pelvic exam, does not face provider bias against it, and is more amenable to task shifting to lower cadres, including frontline community extension workers, as is being done in Ethiopia (for insertion). According to the UN’s 2015 multinational tabulations, no sub-Saharan African country has an IUD prevalence above 4%, only Guinea-Bissau and Kenya have levels above 3%, and the majority of countries have levels below 1%.^7^ By contrast, the implant’s prevalence is considerably higher: 11% in Kenya, 9% in Malawi, 8% in Burkina Faso, 7% in Ethiopia, and 6% in Rwanda.^7^

On the other hand, as Hubacher notes, the IUD has never received the extent of donor support and “buzz” in the international family planning community that has been accorded the implant since the Family Planning 2020 (FP2020) initiative and the donor volume guarantee. And yet, even absent that, the IUD’s share of modern method use in 2014–2015 surveys conducted by Performance Monitoring and Accountability 2020 (PMA2020) is 7% in Kenya,^14^ 4% in Uganda,^15^ 3% in Ethiopia,^16^ and 2% in Burkina Faso,^17^ confirming Hubacher’s point that some sub-Saharan African women will choose an IUD when it is made accessible.

## 5. Complexity of Introducing a New Method

Introduction of a new contraceptive method, with plans for its wider adoption and scale-up, is a complex process that requires considerable and sustained work.^18^ In addition to simple provision of the LNG IUS commodity by donors, introduction would require attending to many aspects of the health system: regulatory requirements; supply chain and logistics management; public- and private-sector service delivery policies and processes; counseling and skills training (the LNG IUS requires a different insertion technique than the Cu T IUD); side effects management; quality assurance; client perspectives and knowledge; provider perspectives and work situations; and community outreach and engagement.

## HOW THEN TO ACHIEVE THE FULL POTENTIAL FOR THE LNG IUS?

In our view 5 things will be required for the LNG IUS to become widely accessible and used:

1. A commodity price comparable to that of contraceptive implants.
2. An introduction and ultimate scale-up plan that addresses the many considerations we’ve laid out above (assuming secure supply of needed LNG IUS devices).
3. Identifying, supporting, and otherwise “nurturing” a nucleus of “champions” to become advocates for the method itself and for its introduction at policy, program, system, and service provider levels.
4. Ensuring that the introduction effort is visible to policy makers and opinion leaders in the relevant government ministries, teaching and training institutions, service delivery systems, and professional organizations (medical, midwifery, and nursing), as well as to potential clients.
5. Robust outreach and demand creation efforts that include emphasizing the LNG IUS’s non-contraceptive benefits in reducing menstrual bleeding, i.e., it is not “just another IUD.”

Achieving scale-up of demand, availability, and access is a longer-term proposition, but one that should be initiated now. Well-conceived, adequately resourced, and well-implemented introduction should ideally start in several countries using import waivers where necessary to acquire the product in places where it is not currently registered. In order to facilitate that start, we describe LNG IUS product options and some of the pathways forward.

### The ICA Foundation: Source of Limited Quantities of Free Product

The legacy LNG IUS product, Mirena, currently sells for well upwards of $400 in the United States, which would be prohibitively expensive for public-sector provision in low-income countries. However, an LNG IUS device (with the same amount of hormone and mechanism of action as Mirena) is available free of charge from the ICA Foundation, a partnership between Population Council and Bayer Healthcare Pharmaceuticals.^19^ (One of this paper’s authors, RJ, serves as an Advisor to the ICA Foundation Board.) Upon request, the ICA Foundation provides free LNG IUS devices to governments, NGOs, and multilateral organizations. From 2004–2015, the Foundation donated more than 70,000 LNG IUS devices to 28 countries, 9 of them in sub-Saharan Africa (including for the introduction efforts in Ghana and Kenya that Hubacher describes^1^^,^^13^). (Information on how to access the LNG IUS can be found at http://www.ica-foundation.org/About_the_Programs/Application_for_New_Projects/.)

The ICA Foundation provides LNG IUS devices free of charge to governments, NGOs, and multilateral organizations.

The ICA Foundation's donations per country have typically been small, largely because the Foundation is unable to provide resources to support other needed aspects of wider introduction, e.g., the costs of shipment, registrations, or waivers, distribution and supply chain management, counseling and skills training, and demand creation. Thus, in the long run, this foundation might not be the best vehicle for a large scale-up of LNG IUS service delivery to tens of thousands (or more) interested clients. However, donations as large as 5,000 to 10,000 units could possibly be provided annually for initial scale-up efforts, concentrating such donations in 3 to 4 low-income countries that have demonstrated interest and with the capacity for larger-scale introduction than has yet occurred.

### Liletta: The New Kid on the Block

Liletta, a low-cost alternative to the legacy product, is being made available by Medicines360.^20^ Approved by the US Food and Drug Administration (FDA) in February 2015, Liletta (known outside the United States as Levosert) contains the same amount of levonorgestrel as Mirena (52 mg), has the same mechanism of action, and releases the same small amount of hormone (20 μg/day) continuously into the uterus. Liletta is currently approved for 3 years of use, with ongoing trials underway to extend its labeled duration of effective use to at least 5 years. (Mirena is approved for 5 years of use.) Liletta does not yet have regulatory approval in low-income countries, nor does Medicines360 have a presence there, so linkage with donors and implementing partners would be essential for registration (or waiver), purchase, introduction, and scale-up—and it could be introduced in these countries now, under a research framework.

## THE WAY FORWARD: AN INITIAL “LEARNING” INTRODUCTION INITIATIVE

Although ultimate scale-up of the LNG IUS is a formidable goal, with substantial funding and implementation challenges, we believe the timing is right for smaller, well-focused introduction/demonstration activities to be launched now. These could assess the LNG IUS’s potential among donors, programs, providers, and clients alike. Such initial projects could access the free LNG IUS commodities now available from the ICA Foundation. Alternatively, devices soon to be available via Medicines360 could be used (as could products from other manufacturers, assuming comparable low commodity cost).

The time is right to launch small LNG IUS demonstration activities in a limited number of countries to assess the method’s potential in low-income countries.

A key part of the introduction strategy would be to support activities in high-quality service delivery situations favorable to IUD provision, such as those using family planning-dedicated providers, mobile outreach service delivery, or social franchising, and with free or very low-cost service provision to clients. On the demand side, having careful evaluation of client acceptability, provider embrace, and method uptake and continuation would be essential in the context of wide contraceptive choice. Mindful of the Cu T’s difficulties, the LNG IUS should be framed as a “new” method, widely used and popular in high-income countries of Europe and North America. Along with high effectiveness and convenience, reduced bleeding and amenorrhea should be specifically promoted as potential advantages, with strong qualitative research to assess that messaging.

In Zambia, the dedicated provider approach, which entailed placement of 18 LARC providers in busy, urban-based, public-sector facilities, resulted in provision of 11,000 Cu T IUDs (and 22,000 implants) in 14 months.^21^ Use of dedicated providers and mobile service delivery over a 3-year span in Malawi led to uptake of more than 130,000 female sterilization procedures (generally more costly and time-consuming to provide than IUDs).^22^ In both situations, services were provided free of cost to clients, and the activities were conducted by an NGO in private-public partnership with the Ministry of Health, both important factors in such large uptake of clinical methods in low-resource settings. The new WHO guidance allowing immediate postpartum provision of hormonal IUDs (and implants)^12^ and the almost universal (and largely unmet) demand for contraception in the 0- to 1-year postpartum period^23^ increases the potential importance of linking this introduction effort to busy maternity settings. These demonstration projects would need to address many of the implementation and scale-up requirements we have outlined earlier.

### What About Price?

Price and aggregate commodity cost remain an unresolved sticking point to scale-up, as a 10- to 30-fold cost differential between the LNG IUS and Cu T IUD would understandably represent a large hurdle for policy makers and program funders. Securing a concentrated supply of LNG IUS devices in several countries via the free devices from the ICA Foundation could be a solid, albeit small, start. Hopefully, the imminent availability of Liletta as well as lower cost LNG IUS devices from other manufacturers will foster competition, and commodity cost will accordingly come down substantially. If initial introduction efforts are successful and quantities increase, that could also further decrease cost. And as with contraceptive implants, approaches like a volume guarantee could provide a major boost.

## CONCLUSION

Under the conditions described above, the LNG IUS—such a highly beneficial, convenient, and potentially popular contraceptive—could become as widely chosen by women in sub-Saharan African countries as it is now by women in high-income countries of Europe and North America. Moreover, it seems likely that these efforts would also improve use of copper IUDs as well, as was the case in Kenya.^13^ Then the IUD would truly be “revitalized,” and women would have more highly effective options in the countries where we work.

In light of the LNG IUS’s considerable game-changing potential, and mindful of these qualifications and amplifications to Hubacher’s call to action, let’s mount a vigorous, well-focused, and multifaceted effort, now.
